# Gene expression profiles in rat mesenteric lymph nodes upon supplementation with Conjugated Linoleic Acid during gestation and suckling

**DOI:** 10.1186/1471-2164-12-182

**Published:** 2011-04-11

**Authors:** Elisabet Selga, Francisco J Pérez-Cano, Àngels Franch, Carolina Ramírez-Santana, Montserrat Rivero, Carlos J Ciudad, Cristina Castellote, Véronique Noé

**Affiliations:** 1 Department of Biochemistry and Molecular Biology, School of Pharmacy, University of Barcelona, Barcelona, Spain; 2 Department of Physiology, School of Pharmacy, University of Barcelona, Barcelona, Spain; 3 CIBER in Epidemiology and Public Health (CIBERESP), Barcelona, Spain; 4 Ordesa Group, Research Department, Scientific Park of Barcelona, Barcelona, Spain

## Abstract

**Background:**

Diet plays a role on the development of the immune system, and polyunsaturated fatty acids can modulate the expression of a variety of genes. Human milk contains conjugated linoleic acid (CLA), a fatty acid that seems to contribute to immune development. Indeed, recent studies carried out in our group in suckling animals have shown that the immune function is enhanced after feeding them with an 80:20 isomer mix composed of c9,t11 and t10,c12 CLA. However, little work has been done on the effects of CLA on gene expression, and even less regarding immune system development in early life.

**Results:**

The expression profile of mesenteric lymph nodes from animals supplemented with CLA during gestation and suckling through dam's milk (Group A) or by oral gavage (Group B), supplemented just during suckling (Group C) and control animals (Group D) was determined with the aid of the specific GeneChip^® ^Rat Genome 230 2.0 (Affymettrix). Bioinformatics analyses were performed using the GeneSpring GX software package v10.0.2 and lead to the identification of 89 genes differentially expressed in all three dietary approaches. Generation of a biological association network evidenced several genes, such as connective tissue growth factor (Ctgf), tissue inhibitor of metalloproteinase 1 (Timp1), galanin (Gal), synaptotagmin 1 (Syt1), growth factor receptor bound protein 2 (Grb2), actin gamma 2 (Actg2) and smooth muscle alpha actin (Acta2), as highly interconnected nodes of the resulting network. Gene underexpression was confirmed by Real-Time RT-PCR.

**Conclusions:**

Ctgf, Timp1, Gal and Syt1, among others, are genes modulated by CLA supplementation that may have a role on mucosal immune responses in early life.

## Background

Food components play a role in influencing, either directly or indirectly (through hormonal regulation), the expression of genes encoding for proteins involved in energy metabolism, cell differentiation and growth and immune responses. More specifically, diet exerts diverse effects on the development of the immune system, even at the level of gene regulation [1]. It is known that polyunsaturated fatty acids (PUFAs) can modulate the expression of a variety of genes encoding for cytokines, adhesion molecules, and inflammatory proteins [2,3]. This fact seems to be very important during early life, since docosahexanoic and arachidonic acids were reported to participate in the development of the neonate immune system, although their proportion among total fatty acids in human breast milk is very low [4]. Human milk contains PUFAs such as conjugated linoleic acid (CLA), among others, that seem to contribute to immune development [5-8].

CLA is a class of positional and geometric conjugated dienoic isomers of linoleic acid, among which, cis9,trans11 (c9,t11) predominate, accounting for 83% to 100% of total CLA present in milk [5-7], whereas trans10,cis12 (t10,c12) exist in lower proportion [9,10]. However, it is the intake of food of ruminant origin which determines the total concentration of CLA in dam's milk [7]. Many other beneficial physiological effects have also been attributed to CLA, including reduced body fat, and inhibition of carcinogenesis, atherosclerosis, and diabetes [11-13].

Existing data regarding the effects of CLA on the immune system show great variability, mainly due to differences in the animal species used, the length of the supplementation period, and the differences in the isomer mixtures used in the experimental approach. In this direction, recent studies in suckling animals showed that the immune function is enhanced after feeding with an 80:20 isomer mix of c9,t11 and t10,c12 CLA [14,15]. Specifically, sera IgG concentration and IgM in vitro production by splenocytes are increased after CLA supplementation during suckling. CLA downmodulatory effects on lymphoproliferation were only observed after an extra week of diet [16]. The immune effects of CLA were also described in adult rats receiving this mixture since pregnancy [17]. However, little work has been done on the effects of CLA on gene expression, and even less regarding the development of the immune system in early life.

Nutritional genomics is a result of the genetic revolution experienced over the past 10 years. Nutrigenomics deals with the interactions between dietary components and the genome and the resulting changes in proteins and other metabolites. On the other hand, nutrigenetics aims to understand the gene-based differences in response to dietary components and to develop nutraceuticals that are the most compatible with the health status of individuals based on their genetic makeup [18]. The number of successful examples of transcriptome, proteome, and metabolome profiling as tools for evaluating the cellular responses to nutrients and identifying their molecular targets, has grown significantly. The use of high-density microarrays is a useful approach to estimate correlations among genes, which in turn can become the basis of transcriptional networks. The availability of microarrays for a number of model systems allows the quantification of relative transcript abundances in a comprehensive fashion. Despite the relatively simplistic nature of correlation measurements, they reflect an integrative view of gene-gene interactions in any given system, pointing out general structure characteristics of transcriptional interaction networks [19].

Additionally, nutrigenomic approaches have been undertaken to get further insight on the molecular understanding of mechanisms triggered by nutritional interventions. Diets enriched in different long-chain polyunsaturated fatty acids (LC-PUFAs) have been tested in rat nutritional intervention models. One report revealed steaoryl-CoA desaturase as an enzyme target for an arachidonate-enriched diet [20]. In another study, Berger et al. [21] looked at transcriptional effects of these LC-PUFA-enriched diets on murine hepatic and hippocampal gene expression. Additionally, the beneficial effect of LC-PUFAs was assessed by a nutrigenomics experiment designed to understand the mechanisms by which these lipids induce and control gene signalling involved in carcinogenesis [22].

With all these concepts in mind and based on previous studies, we hypothesized that CLA intake during developmental phases would exert some influencing effect, among others, on genes involved in the regulation of the immune system. The aim of our study was the evaluation, by using whole genome microarrays, of the effects of dietary supplementation with an 80:20 isomer mix of c9,t11 and t10,c12 CLA, on mesenteric lymph nodes (MLN) gene expression, during gestation and/or suckling. The list of common genes differentially expressed in the three dietary interventions was used to construct a Biological Association Network (BAN). This approach allowed us to obtain a global view of gene expression in MLN, formed by a collection of nodes with different degrees of interrelationship, that could be used to explain the molecular mechanisms triggered by CLA.

## Results

### Effect of CLA supplementation on rat MLN gene expression profiles

**Figure 1:**
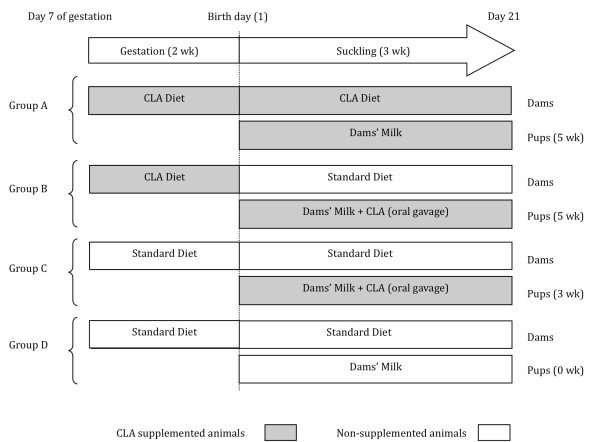
**Experimental design**. Total period of CLA supplementation (TPS) is shown in parentheses for each group of animals. Pups from dams fed with 1% CLA diet during the last two weeks of gestation and throughout the suckling period constituted Group A. Pups from dams fed only during the last two weeks of gestation with a 1% CLA diet and receiving CLA by daily oral gavage throughout the suckling period represented Group B. Pups from dams fed with a standard diet during the last two weeks of gestation and suckling and receiving CLA by daily oral gavage throughout the suckling period corresponded to Group C. Pups from dams fed with a standard diet throughout the study, constituted the reference diet group our Group D.

Wistar rats were subjected to a dietary supplementation during gestation and/or suckling with an 80:20 isomer mix of c9,t11 and t10,c12 CLA (Figure 1). CLA transfer was confirmed at day 21, when the proportion of CLA present in pup's plasma was around two times higher in groups A and B than that in group C, while values on reference group were very low [15]. At day 21, MLN were obtained and total RNA was prepared. The expression profile of MLN from control animals and supplemented with CLA was determined with the aid of the specific GeneChip^® ^Rat Genome 230 2.0, which includes more than 28,000 rat genes. Data from these microarrays were uploaded in the database repository of Gene Expression Omnibus (GEO, [23]) and can be accessed through series accession number GSE23004. Upon normalization and statistical filtering using GeneSpring GX software package v10.0.2, lists of differentially expressed genes by 2-fold were built as described in Methods, and presented as additional files 1, 2 and 3. CLA supplementation during gestation and suckling (groups A and B) modulated the expression of 1332 genes whereas the dietary supplementation only during suckling modulated the expression of 517 genes. Moreover, CLA supplementation during gestation and suckling through dam's milk (group A) decreased the expression of 1001 genes and up-regulated 103 genes in MLN. The dietary supplementation during gestation and suckling by oral gavage (group B) decreased the expression of 257 genes and up-regulated 371 genes, whereas supplementation only during suckling (group C) decreased the expression of 271 genes and up-regulated 351 genes in MLN.

### Identification of common genes modulated by CLA supplementation

**Figure 2:**
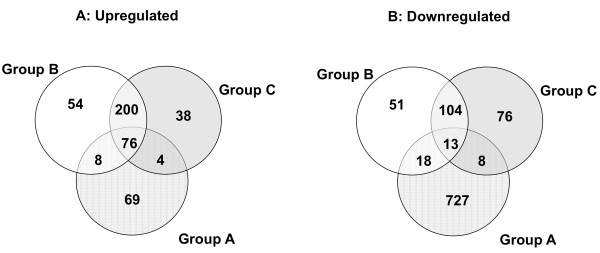
**Venn Diagram of differentially expressed genes**. Venn diagrams in GeneSpring GX were used to compare the lists of genes differentially expressed by 2-fold (p < 0.05) in each of the three experimental groups of animals. The diagrams show the number of genes that belong to each of the individual lists, the genes in common between each pair of lists and the genes in common among all three lists (in the center of the representation) for each type of regulation (A: Upregulation and B: Downregulation).

Venn diagrams were used to compare the lists of 2-fold differentially expressed genes obtained for the three experimental groups of animals (Figure 2). Lists of upregulated and dowregulated genes were compared separately in order to find the same expression patterns between the dietary interventions compared (e.g. genes upregulated in both). Experimental groups A and B shared 84 upregulated and 31 downregulated genes, groups A and C displayed 80 upregulated and 21 downregulated genes in common and 276 upregulated and 117 downregulated genes were found in common for both groups B and C. A total of 89 genes were found to be differentially expressed in all three conditions due to CLA supplementation (76 up plus 13 down). These 89 genes are presented as Table 1. We also analyzed global changes in gene expression independently of the type of regulation. We observed that 34 genes displayed downregulation in group A (CLA transfer through milk) and upregulation in groups B/C (CLA transfer by oral gavage) (Data not shown). This behavior suggests a role for the way of CLA administration in modulating gene expression.

**Table 1:** Common genes among the three experimental groups supplemented with CLA.

Gene Symbol	Description	Gene Ontology Biological Process	Ratio A	Ratio B	Ratio C
Actg2	actin, gamma 2, smooth muscle, enteric		70.79 D	97.48 D	105.85 D

Cnn1	calponin 1, basic, smooth muscle	actin cytoskeleton organization	17.18 D	20.13 D	19.90 D

Myh11	myosin, heavy chain 11, smooth muscle	smooth muscle contraction	16.49 D	16.57 D	19.68 D

Pcp4	Purkinje cell protein 4		12.37 D	12.81 D	12.94 D

Tpm2	tropomyosin 2	regulation of ATPase activity	7.89 D	7.26 D	6.84 D

Krt19	keratin 19	response to estrogen stimulus	7.78 D	5.59 D	9.12 D

Tpm1	Tropomyosin 1, alpha	muscle contraction	7.38 D	8.09 D	8.27 D

Cfb	complement factor B	complement activation	7.11 D	4.99 D	7.82 D

Acta1	actin, alpha 1, skeletal muscle	cell growth	7.10 D	7.54 D	7.18 D

Gal	galanin prepropeptide	inflammatory response	6.38 D	7.03 D	7.72 D

Rnase4	ribonuclease, RNase A family 4	proton transport	5.38 D	2.53 D	4.06 D

Upk1b	uroplakin 1B	epithelial cell differentiation	5.36 D	4.17 D	5.51 D

Rnase4	ribonuclease, RNase A family 4	proton transport	5.36 D	2.38 D	3.58 D

Mylk	myosin light chain kinase		5.04 D	4.11 D	3.23 D

Tpm1	tropomyosin 1, alpha	muscle contraction	4.97 D	5.34 D	5.80 D

Tnfaip6	tumor necrosis factor alpha induced 6	cell adhesion	4.94 D	3.35 D	4.39 D

Klhl23	kelch-like 23		4.62 D	2.99 D	2.93 D

Tnfrsf11b	tumor necrosis factor receptor 11b	signal transduction	4.37 D	2.02 D	2.46 D

Fhl1	four and a half LIM domains 1	cell differentiation	4.22 D	2.56 D	2.73 D

Synm	synemin, intermediate filament protein		3.96 D	3.69 D	4.74 D

Tmem100	transmembrane protein 100		3.76 D	3.06 D	3.71 D

Smoc2	SPARC related modular calcium binding 2	cell-substrate adhesion	3.68 D	5.14 D	7.01 D

Pln	phospholamban	calcium ion transport	3.68 D	3.66 D	3.89 D

Tm4sf1	transmembrane 4 L six family member 1		3.61 D	2.09 D	2.35 D

Acin1	apoptotic chromatin condensation inducer 1	chromosome condensation	3.59 U	3.09 U	2.69 U

Grem2	gremlin 2, cysteine knot superfamily, homolog	BMP signaling pathway	3.52 D	3.38 D	4.83 D

Pik3c2b	phosphoinositide-3-kinase, class 2, beta	cell communication	3.45 U	3.42 U	2.95 U

Fbxl22	F-box and leucine-rich repeat protein 22		3.36 D	3.72 D	3.67 D

Nupr1	nuclear protein 1	acute inflammatory response	3.31 D	3.00 D	3.01 D

Nov	nephroblastoma overexpressed gene	regulation of cell growth	3.27 D	3.10 D	3.48 D

Scg2	secretogranin II	MAPKKK cascade	3.22 D	3.16 D	3.15 D

Rbm5	RNA binding motif protein 5	nuclear mRNA splicing	3.14 U	3.99 U	4.04 U

Tpm1	tropomyosin 1, alpha	muscle contraction	3.11 D	3.29 D	3.27 D

Tpm1	tropomyosin 1, alpha	muscle contraction	3.11 D	4.63 D	4.57 D

Bzrap1	benzodiazapine receptor associated protein 1		3.08 U	3.61 U	3.25 U

Zeb2	Zinc finger E-box binding homeobox 2	regulation of transcription	3.07 U	5.80 U	2.06 U

Adamts1	ADAM metallopeptidase with thrombospondin 1	proteolysis	3.01 D	2.26 D	2.34 D

Myl9	myosin, light chain 9, regulatory		3.01 D	5.94 D	5.54 D

Gap43	growth associated protein 43	regulation of cell growth	2.98 D	3.34 D	3.22 D

Aldh1a1	aldehyde dehydrogenase 1 family, A1	metabolic process	2.94 D	2.77 D	2.91 D

Krt8	keratin 8	apoptosis	2.93 D	3.97 D	3.54 D

Gstm5	glutathione S-transferase, mu 5	metabolic process	2.86 D	3.14 D	2.81 D

Sh3bgr	SH3 domain binding glutamic acid-rich		2.85 D	3.32 D	3.40 D

Prph	peripherin	cytoskeleton organization	2.81 D	3.59 D	4.12 D

Ctgf	connective tissue growth factor	cartilage condensation	2.80 D	3.59 D	5.12 D

Sparcl1	SPARC-like 1		2.74 D	2.00 D	2.65 D

Pgm5	phosphoglucomutase 5	carbohydrate metabolism	2.74 D	3.86 D	4.72 D

Klhl23	kelch-like 23		2.74 D	2.26 D	2.16 D

Fhl1	four and a half LIM domains 1	cell differentiation	2.73 D	2.74 D	3.13 D

Acta2	smooth muscle alpha-actin	muscle contraction	2.70 D	4.63 D	2.86 D

Msln	mesothelin	cell adhesion	2.69 D	2.58 D	3.63 D

Sstr3	somatostatin receptor 3	signal transduction	2.67 U	3.11 U	2.90 U

Ppp1r14a	protein phosphatase 1, regulatory subunit 14A	phosphorylation	2.65 D	4.99 D	4.83 D

Timp1	TIMP metallopeptidase inhibitor 1	cell activation	2.64 D	4.04 D	4.54 D

Bcl2l2//Pabpn1	Bcl2-like 2//poly(A) binding protein nuclear 1	apoptosis	2.64 U	2.40 U	2.40 U

Ash1l	Ash1 (absent, small, or homeotic)-like		2.58 U	3.43 U	3.85 U

Mylk	myosin light chain kinase		2.57 D	3.10 D	3.94 D

Adh1	alcohol dehydrogenase 1	retinoid metabolism	2.52 D	2.52 D	2.56 D

Ptgis	prostaglandin I2 synthase	prostaglandin biosynthesis	2.48 D	2.92 D	3.33 D

Stmn2	stathmin-like 2	intracellular signaling	2.45 D	2.40 D	2.33 D

Gpd1	glycerol-3-phosphate dehydrogenase 1	gluconeogenesis	2.43 D	2.40 D	2.79 D

Pdlim3	PDZ and LIM domain 3	actin filament organization	2.42 D	2.33 D	2.50 D

Cgnl1	cingulin-like 1		2.41 D	2.01 D	2.15 D

Pla2g2a	phospholipase A2, group IIA	phospholipid metabolism	2.41 D	2.93 D	3.05 D

Ikzf2	IKAROS family zinc finger 2		2.39 U	5.10 U	4.82 U

Rgs4	regulator of G-protein signaling 4	signal transduction	2.39 D	2.27 D	2.55 D

Argbp2	Arg/Abl-interacting protein ArgBP2	intracellular signaling	2.36 D	2.30 D	2.45 D

Fgf13	fibroblast growth factor 13	MAPKKK cascade	2.35 D	2.80 D	2.86 D

Igf2	insulin-like growth factor 2	cell proliferation	2.30 D	4.82 D	6.01 D

Syt1	synaptotagmin I	transport	2.29 D	2.46 D	2.30 D

Hspb1	heat shock protein 1	response to heat	2.24 D	3.69 D	5.15 D

Gja5	gap junction protein, alpha 5	cell communication	2.24 D	3.04 D	2.93 D

Schip1	schwannomin interacting protein 1		2.23 D	2.92 D	2.80 D

Efemp1	EGF-containing fibulin-like extracellular matrix 1		2.23 D	2.41 D	3.39 D

Cxcr7	chemokine (C-X-C motif) receptor 7	signal transduction	2.22 D	2.24 D	3.13 D

Phemx	pan hematopoietic expression		2.21 U	2.56 U	2.61 U

Gpc3	glypican 3	regulation of growth	2.20 D	3.02 D	4.39 D

Leng8	leukocyte receptor cluster member 8		2.20 U	2.34 U	2.10 U

Crispld2	cysteine-rich secretory protein LCCL domain containing 2	lung development	2.20 D	3.82 D	3.77 D

Tagln	transgelin	cytoskeleton organization	2.20 D	3.49 D	3.54 D

Grb2	growth factor receptor bound protein 2	MAPKKK cascade	2.19 U	2.42 U	2.25 U

Parva	parvin, alpha	cell adhesion	2.16 D	2.04 D	2.30 D

Hist1h4b	histone cluster 1, H4b	nucleosome assembly	2.15 U	2.44 U	2.08 U

Des	desmin		2.12 D	3.69 D	4.05 D

Cryab	crystallin, alpha B	glucose metabolic process	2.09 D	3.37 D	3.18 D

Enpp3	ectonucleotidepyrophosphatase phosphodiesterase 3	phosphate metabolism	2.05 D	2.21 D	2.52 D

Bag2	Bcl2-associated athanogene 2	apoptosis	2.04 D	3.04 D	3.12 D

Wfdc1	WAP four-disulfide core domain 1	regulation of cell growth	2.01 D	2.66 D	2.80 D

Cd9	CD9 molecule	cell adhesion	2.01 D	2.24 D	2.12 D

The lists of genes differentially expressed by 2-fold with a p-value < 0.05 obtained for each experimental group were compared using Venn Diagrams in GeneSpring GX software v 10.0.2. The table shows the list of genes differentially expressed in all three conditions, and includes the Gene Symbol for all genes, their associated description and one of the Gene Onthology categories to which the genes belong according to GeneSpring GX. The ratio columns correspond to the absolute fold change in expression for the genes in each experimental group (A, B or C) relative to the control group (D) and the type of regulation (U, upregulation; D, downregulation)

### Detection of gene nodes upon BAN generation

**Figure 3:**
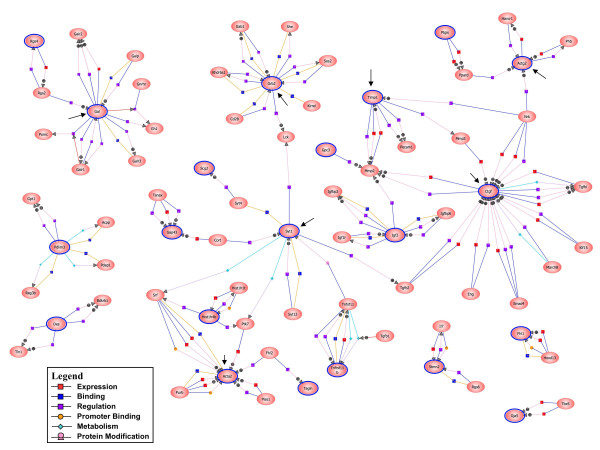
**BAN of differentially expressed genes**. The list of common genes among the three experimental groups of animals was the starting point to construct a BAN using the Pathway Architect software within GeneSpring GX. Expanded networks were constructed by setting an advanced filter that included the categories of binding, expression, metabolism, promoter binding, protein modification and regulation (see inset legend). Only proteins are represented. Genes present in the original list of common genes are encircled in blue, whereas the other genes were added by the software from the interactions database. The BAN presented shows some highly interconnected node genes that were object of further studies (pointed with arrows).

A BAN was generated as described in Methods with the list of differentially expressed genes in common among the three groups of animals. This type of graphical representation evidenced several genes, such as connective tissue growth factor (Ctgf), tissue inhibitor of metalloproteinase 1 (Timp1), galanin (Gal), synaptotagmin 1 (Syt1), growth factor receptor bound protein 2 (Grb2), actin gamma 2 (Actg2) and smooth muscle alpha actin (Acta2), as highly interconnected nodes of the resulting network (Figure 3).

### Validation of node-genes selected from the BAN

**Figure 4:**
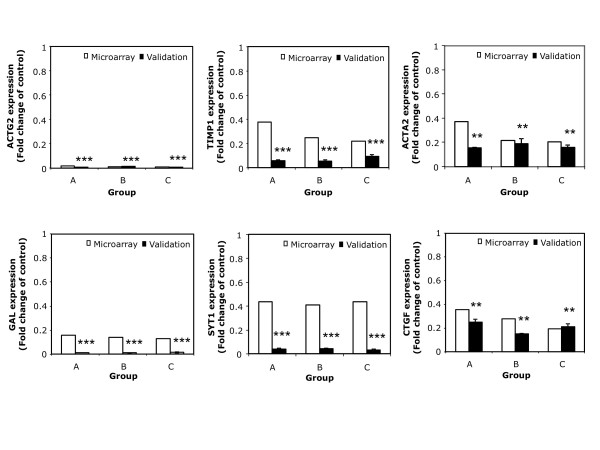
**mRNA levels of selected node genes**. The mRNA levels of the indicated genes were determined for the four groups of animals by RT-Real Time PCR as described in Methods. Empty bars represent the mRNA levels for each specific gene as determined in the microarray experiments, filled bars represent the mRNA levels for each specific gene as determined by RT-Real Time PCR. ** p < 0.01 and *** p < 0.001.

We proceeded to validate the differential expression of the node-genes to verify the changes in their mRNA levels. Real-time PCR offers a non hybridization-based detection and was chosen as complementary to microarrays. The genes selected from the BAN representation were analyzed by RT-Real time PCR under the same experimental conditions as for the microarray analyses. Results are shown in Figure 4, where the levels of mRNA determined both in the microarrays and by RT-Real Time PCR for each gene in each experimental group are represented. In MLN, the mRNA levels for Gal and Actg2 were decreased by around 99%, and those of Timp1 and Syt1 decreased by 90-95% upon CLA administration in all groups of animals tested (p < 0.001). Treatment with CLA also decreased mRNA expression of Ctgf and Acta2 by around 80% (p < 0.01). Grb2 showed no changes in gene expression when determined by RT-Real Time PCR (p > 0.05, data not shown), at variance with the data obtained from the microarrays. These results confirmed the RNA data obtained in the screening performed using the microarrays for all the selected genes with the exception of Grb2.

## Discussion

In this work, we analyzed the gene expression profile of MLN cells from suckling rats receiving an 80:20 c9,t11:t10,c12 CLA mix during early life. This particular mixture enriched with the c9,t11 isomer was used due to its resemblance with that in human breast milk [5]. Previous studies have demonstrated that this mix modulates immune development in suckling rats receiving it during gestation/suckling [15]. The main effect of this type of diet was found regarding immunoglobulin production at systemic and intestinal level [14,15]. However, the precise mechanism of action of this PUFA was not established. Thus, we used microarrays to get further insight in the effects of CLA supplementation on MLN gene expression. A large number of genes were modulated when CLA was administered during gestation and suckling (groups A and B, 5 wk). However, the use of Venn Diagrams evidenced that many genes were differentially expressed in both groups B and C, and less common genes were found between group A and the others. This behavior could be due to the different nutritional intervention that was performed. In the group A, CLA was added to the diet of the dams during suckling period and therefore CLA was incorporated in breast milk and transferred to the suckling pups. However, in groups B and C, CLA was directly administered by oral gavage to the suckling pups. This fact influences the proportion between the two CLA isomers that get to pups, as the ratio of c9,t11 CLA/t10,c12 CLA in pups's plasma was 83:14 for group A, but 93:7 for group B and 94:6 for group C as previously described [15].

The list of differentially expressed genes in the three conditions was used to generate a BAN. Several node genes, namely Ctgf, Timp1, Gal, Syt1, Actg2 and Acta2, were selected from this BAN, and changes in their mRNA expression were confirmed by RT-Real time PCR. Some of these node-genes may explain the behavior of CLA as immune modulator during early age.

Connective tissue growth factor (Ctgf) is a secreted extracellular matrix-associated protein that modulates many cellular functions, including proliferation, migration, adhesion, and extracellular matrix production, and it is involved in several biological and pathological processes [24]. Dietary supplementation with CLA reduced mRNA levels of Ctgf in MLN in our three experimental approaches, indicating that downregulation of Ctgf is independent of the length and via of CLA supplementation. Ctgf is mainly regulated at the transcriptional level by bioactive lipids, as well as TGFβ and downstream mitogen-activated protein (MAP) kinase signaling [25].

We have previously reported that young (28-day-old) and adult animals fed with the same CLA mixture showed a reduction in the proliferation activity of spleen and MLN cells [16,17]. Given that Ctgf promotes DNA synthesis in chondrocytes, osteoblasts and fibroblasts [26], the observed decrease in proliferation could be mediated through Ctgf downregulation.

Tissue inhibitors of metalloproteinases (Timp) are multifunctional proteins including four members (Timp1-4). Timp1 is expressed and secreted by a variety of cell types and is present in most tissues [27,28]. Timp1 is able to promote cell growth, and its expression levels inversely correlate with the susceptibility to induction of apoptosis (reviewed in [29]).

Until now, two studies have reported different effects of CLA on Timp1 expression. On one hand, 1% CLA diet in a mice model of metastatic mammary tumor increased mRNA and protein levels of Timp1 [30], whereas an *in vitro *study using pure CLA isomers (c9,t11 CLA and t10,c12 CLA) on a macrophage cell line did not modify Timp1 gene expression [31]. Thus, the effect of CLA on Timp1 expression may depend on the tissue microenvironment and the experimental approach used. In our study, CLA administration reduced mRNA levels for Timp1 in all three groups of animals. As Timp1 may stimulate cell proliferation by using several distinct signalling pathways, such as MAPK, the inhibitory effect of CLA on this gene may reduce lymphoproliferation. Thus, both Timp1 and Ctgf would play a role in a same signaling pathway that could explain the downmodulation of the lymphoproliferative response observed in 28-day-old animals fed with CLA from birth [16].

The neuropeptide Galanin (Gal) was also downregulated at the mRNA level by early CLA administration, independently of length, duration and via of supplementation. This peptide exerts a wide range of effects, not only in the central nervous system [32] but also in the enteric nervous system [33]. Although Gal is mainly produced by neurons, it has been described that lymphocytes and macrophages can also synthesize it at a lower level [33]. A clear effect of neuropeptides such as somatostatin (SOM) or vasoactive intestinal peptide (VIP), including gut-associated immune system (GALT), has been already reported on the immune system [34]. When these neuropeptides are found in the intestine, they seem to affect the proliferation, differentiation and function of immune cells [33]. Thus, the effect of CLA on Gal expression could explain the immunoenhancing effects of CLA on developing GALT [17].

There is no data available on the effects of Gal on lymphoproliferation. SOM and VIP are mainly inhibitors of lymphocyte proliferation, apparently by reducing interleukin-2 production [34], whereas calcitonin-gene-related-peptide (CGRP), substance P (SP) or met-enkephalin stimulate lymphoproliferation [35,36]. Gal-mediated effects through its receptor Galr1 led to antiproliferative effects, whereas those through Galr2 and Galr3 were proliferative. All three receptors are expressed in immune cells [37]. When the same CLA mix was administered to young rats supplemented during suckling [16] or until adult age [17], a downmodulatory activity on proliferation in both spleen and MLN was observed, suggesting that this effect may be mediated in part by modulation of Gal expression. Thus, CLA is able to reduce Gal promoting activity on lymphoproliferation, indicating that Gal would be acting mainly through its Galr2 and Galr3 receptors in that environment.

During early life CLA is able to enhance the production of immunoglobulins [15] and more specifically IgA in the intestine [14]. Enhancement of intestinal IgA concentration by CLA could be due to the decrease of Gal expression in MLN, as it is the case for SOM in murine spleen, Peyer patches and MLN [38]. Moreover, as neuropeptides influence cytokine production, downregulation of Gal by CLA may be the responsible effector of the changes in cytokine concentrations found in previous studies [14,15]. However, the effect on Gal found here in early life, and their parallel immune effects, may not be present at older ages, since the maturation state of lymphocytes seem to affect the response to neuropeptides and cytokines due to their differential neuropeptidergic receptor expression [33]. This fact is in agreement with the effect described for CLA on developing immune system and immunoglobulin production [15,17], which is not observed later in life.

Synaptotagmins (Syts) constitute a family of proteins that act as Ca^2+-^sensors for regulated exocytosis and endocytosis [39-41]. Synaptotagmin proteins were first detected in the nervous system, but their expression has also been reported in other tissues. Thus, synaptotagmins have been proposed to be widespread regulatory proteins that confer Ca^2+-^sensitivity in a wide range of fusion events [39]. Indeed, Syts have been described to play a role in the maintenance of plasma membrane integrity [40], in cell resealing [42], in chemotactic cell migration [43], in insulin release from the pancreatic β-cells [44,45], in FGF-1 secretion in response to heat shock [46] and in degranulation of mast cells [47].

CLA supplementation caused downregulation of Syt1 by about 2.3 fold in the three dietary approaches. Syt1 is mainly localized in synaptic vesicles, where it functions as a Ca^2+-^sensor for exocytosis [48]. Indeed, Syt1 has been proposed to play specific roles in synaptic vesicles trafficking, in facilitating docking [49], and in vesicle fusion [50]. Although it has been traditionally proposed to be a neuron-specific isoform, Syt1 has been demonstrated to have important roles in membrane trafficking in non-neural and non-hormonal cells [51,52].

Syt1 was previously associated with CLA supplementation, as its expression was significantly decreased in mice receiving a diet enriched in the t10,c12 CLA isomer compared with c9,t11 CLA [52]. Syt1 downregulation was associated with the presence of hepatic fat, suggesting that changes in expression of Syt1 may indicate that other genes than the traditional are involved in lipid handling in hepatic cells, and probably in MLN. Syt1 has also been shown to play a role in neurotransmitter release [53,54]. As described above, we found the neuropeptide Gal underexpressed upon CLA supplementation. Since it has been described that Gal is secreted by enteric nerves [55], we propose that downregulation of Syt1 upon CLA supplementation would lead to a decrease in Gal secretion, thus enhancing the negative effects that downregulation of Gal would have on lymphoproliferation.

Actins are highly conserved proteins that are involved in various types of cell motility, and maintenance of the cytoskeleton. In vertebrates, three main groups of actin isoforms, alpha, beta and gamma have been identified. The alpha actins are found in muscle tissues and are a major constituent of the contractile apparatus. The beta and gamma actins co-exist in most cell types as components of the cytoskeleton, and as mediators of internal cell motility. There are three α-actins (skeletal, cardiac, and smooth muscle), one β-actin (β-nonmuscle), and two γ-actins (γ-smooth muscle and γ-nonmuscle) [56]. The cytoskeleton constitutes a network that serves to maintain cell shape and to regulate dynamic cellular functions. It provides essential scaffolding for the localization and activation or inhibition of diverse cytoplasmic signaling molecules, as well as anchors for motor proteins that are necessary for intracellular transport and cell division [57,58]. In our microarray experiments we found that expression of smooth muscle γ-actin (Actg2), found in enteric tissues, and α2-actin (Acta2) were decreased upon dietary supplementation with CLA.

Alpha-smooth muscle actin expression is regulated by TGF-β and TNF-α. Changes in alpha-smooth muscle actin, collagen, and fibronectin expression result in decreased contraction and stiffness of collagen matrices [59]. The effect of TGF-β has been related to a conserved TGF-β control element (TCE) within the 5'-region of alpha-smooth muscle actin, also known as smooth muscle cell differentiation marker. TCE could mediate both transcriptional activation and repression in cultured smooth muscle cells through interaction with members of the zinc finger Kruppel-like transcription factor family [60]. On the other hand, it has been reported that TGF-β1 is able to significantly increase Acta2 expression in both normal and cancer associated fibroblasts cultures [61], which are believed to promote tumor growth and progression. Overall, the downregulation of actins upon CLA supplementation would affect both the contractile apparatus and the cytoskeleton with a final effect on cell motility. However its relationship with the development of the immune system remains unclear. Deficiencies in proteins that regulate the cortical actin cytoskeleton have increasingly been associated with immunodeficiency and autoimmune or autoinflammatory disease, indicating a critical role for these regulators in immune response and tolerance (reviewed in [56]).

Besides the modulatory action of CLA found on the above node genes, other genes with importance on the immune system were differentially modulated and would require further studies. This is the case of for the receptors for interleukin-7 (Il7r) and chemokines (i.e. Cxcr7) which are involved in mucosal immune responses and chemotaxis, respectively [62,63].

## Conclusions

In summary, by using rat whole genome microarrays, we determined the changes in gene expression induced by an 80:20 c9,t11:t10,c12 CLA mix in MLN from suckling rats. The generation of a biological association network allowed the identification of specific node genes that might be involved in immune responses. We conclude that Ctgf, Timp1, Gal and Syt1, among others, are gene target candidates modulated by CLA which may explain the effect of this PUFA on mucosal immune responses in early life.

## Methods

### Animals

Wistar rats at 7 days of gestation were obtained from Harlan (Barcelona, Spain) and housed in individual cages under conditions of controlled temperature and humidity in a 12 h:12 h light:dark cycle, with access to food and water *ad libitum*. The rats were monitored daily and allowed to deliver at term. The day of birth was reported as day 1. Pups, unified in litters of ten per lactating dam, had free access to the nipples and rat diet. Animals were daily identified and weighed, and handling was done in the same time range to avoid the influence of biological rhythms. On day 21 (weaning day), pups were anaesthetized with ketamine/xylazine (ketamine 90 mg/kg plus xylacine 10 mg/kg of rat weight) and sacrificed by humanitarian methods. MLN were obtained and were flash-frozen in liquid N_2 _and stored immediately at -80°C until processing. Studies were performed in accordance with the institutional guidelines for the care and use of laboratory animals established by the Ethical Committee for Animal Experimentation of the University of Barcelona and approved by the Catalonian Government (CEEA 303/05, UB/DMA 3242).

### Experimental Design

Animals were randomly distributed in 4 dietary groups, according to total period of CLA supplementation and administration route used in the pups [15]. Total period of CLA supplementation (TPS) is shown in parentheses in the experimental design (Figure 1). Pups from dams fed with a 1% CLA diet during the two last weeks of gestation and throughout the suckling period constituted Group A. These pups received CLA through the dam's milk during suckling (TPS 5 wk). Pups from dams fed only during gestation (two last weeks) with a 1% CLA diet and receiving a daily CLA supplement by oral gavage throughout the suckling period (TPS 5 wk) constituted group B. Pups from dams fed with a standard diet during the two last weeks of gestation and suckling and receiving CLA by daily oral gavage throughout the suckling period (TPS 3 wk) constituted Group C. Group D, pups from dams fed standard diet throughout the study, constituted the reference diet group (TPS 0 wk).

In this later group, CLA levels present in milk samples were very low for the c9,t11-CLA isomer (0.02 ± 0.00% in total milk fatty acids) and undetectable for the t10,c12-CLA isomer [15].

### Dietary CLA supplementation

The standard diet corresponded to the American Institute of Nutrition (AIN)-93G formulation [64], containing 7% soybean oil. A 1% CLA diet was obtained from modified standard flour AIN-513 (Harlan) containing 10 g CLA/kg as previously described [14]. Thus, the supplemented diet contained 6% soybean oil plus 1% CLA oil. The CLA isomer mixture used was 80% c9,t11 and 20% t10,c12 from the total CLA isomers in oil. The 1% CLA diet in suckling animals corresponded to a daily administration of 1.5 mg CLA oil provided/g rat from day 1 to 21. Low-capacity syringes (Hamilton Bonaduz AG) adapted to oral 25- or 23-gauge gavage tubes, 27 mm in length (ASICO) were used for oral administration before and after day 5, respectively. To allow gastric emptying, litters were separated from dams 1 h before oral supplementation [65]. CLA arrival to pups was confirmed by its quantification in 21-day-old plasma; moreover, immunomodulatory action of CLA in these animals by means of evaluation of sera Ig concentration, and *in vitro *Ig production and proliferation by isolated splenocytes was also previously observed [15].

### RNA extraction

Three animals were randomly selected from the 10 that constituted each experimental group. Total RNA from MLN of each animal was prepared using the RNAeasy Lipid Tissue Mini kit (Qiagen) following the recommendations of the manufacturer. Briefly, tissue samples were thawed and homogenized in QIAzol Lysis Reagent. After addition of chloroform, the homogenate was separated into aqueous and organic phases by centrifugation. The aqueous phase was applied to the RNeasy spin column and RNA was eluted in RNase-free water. An additional step of phenol-chloroform extraction and ethanol precipitation was performed to ensure the purity of the RNA samples from MLN. Ribosomal RNA band integrity was assessed on an Agilent BioAnalyzer 2100 using an RNA Nano LabChip (Agilent Technologies).

### Microarrays

Gene expression was determined by hybridization to the GeneChip^® ^Rat Genome 230 2.0 (Affymettrix), that allows the simultaneous analysis of the expression level of over 30,000 transcripts and variants from over 28,000 well-substantiated rat genes. Labeling, hybridization and detection were carried out following the manufacturer's specifications. Triplicate samples were hybridized for each experimental condition.

### Microarray data analysis

Gene expression analysis was carried out with GeneSpring GX v10.0.2 software (Agilent Technologies), using the latest gene annotations available. This software package allows multifilter comparisons using data from different experiments to perform the normalization, generation of restriction lists and the functional classification of the differentially expressed genes. All the samples were normalized against the median of the control samples (Group D). The expression of each gene is reported as the ratio of the value obtained after each condition relative to control condition after normalization of the data. Lists of differentially expressed genes were generated using data from the three independent experiments for each condition. A first filter was applied to select the genes that displayed a p-value of less than 0.05. The output of this analysis was then filtered by fold expression, generating lists of differentially expressed genes by 2-fold for each of the experimental groups. These lists were split in two others of upregulated or downregulated genes. Comparisons of the lists of upregulated genes among them were performed by Venn Diagrams in GeneSpring GX. Lists of downregulated genes were also compared among them using the same approach. This procedure allowed us to find differentially expressed genes that followed the same pattern (e.g. upregulated or downregulated) in common among the experimental conditions.

### Biological Association Networks generation

The list of common genes differentially expressed by 2-fold with a p-value < 0.05 in the tree groups of animals was used to construct a biological association network (BAN) using the Pathway Architect software integrated within GeneSpring GX. Briefly, the Pathway Architect software generates interaction networks starting with the genes in a given list (entities) taking into account the information present in a database of known molecular interactions. The lists correspond to the collection of differentially expressed genes under specific conditions. The database of molecular interactions is composed by more than 1.6 million interactions divided into different classes (binding, regulation, promoter binding, transport, metabolism, protein metabolism and expression). The interactions are extracted from literature using a Natural Language Processing tool run on Medline Abstracts (NLP references), plus those obtained from external curated databases like BIND [66] and MINT [67]. Interactions in the interaction database are scored into 5 different categories: maximum, high, medium, low and minimal. Curated interactions (BIND and MINT sources) get the Maximum quality score as do any interactions which have at least 3 NLP references. Pathway Architect gathers all that information to construct novel views as to how the entities in a list could be interacting with each other, even including entities not present in the original list (neighbors resulting from the expanded interaction). Customized analyses were performed to select relevance interaction networks with an associated high confidence index since such networks are likely to mirror biological significance. One-step expansion (expand network) of the original set of entities with maximum score interaction were then analyzed by setting an advanced filter that included the categories of binding, expression, metabolism, promoter binding, protein modification and regulation. This procedure gives a final view formed by a collection of nodes with different degrees of interrelationship. A number of gene products from the original list that were not significantly connected with the other members or neighbors were removed from the final view.

### RT-Real Time PCR

cDNA was synthesized in a total volume of 20 μl by mixing 1 μg of total RNA, 125 ng of random hexamers (BioTools), in the presence of 75 mM KCl, 3 mM MgCl_2_, 10 mM dithiothreitol, 20 units of RNAsin (Promega), 0.5 mM dNTPs (BioTools), 200 units of M-MLV reverse transcriptase (Invitrogen) and 50 mM Tris-HCl buffer, pH 8.3. The reaction mixture was incubated at 37°C for 60 min. The cDNA product was used for subsequent amplification by Real Time-PCR. The expression levels of outlier genes differentially expressed in the microarrays was determined in an ABI Prism 7000 Sequence Detection System (Applied Biosystems) using 3 μl of the cDNA mixture and the Assays-on-demand Rn00583681_m1 for Gal, Rn00587558_m1 for Timp1, Rn00573960_g1 for Ctgf, Rn00578230_m1 for Grb2, Rn00436862_m1 for Syt1, Rn00563662_m1 for Actg2, Rn01759928_g1 for Acta2 and Rn01432775_m1 for Aprt (all from Applied Biosystems). Aprt mRNA was used as an endogenous control. The reaction was performed following the manufacturers recommendations. Fold-changes in gene expression were calculated using the standard ΔΔCt method.

## Competing interests

The authors declare that they have no competing interests.

## Authors' contributions

CC, FJPC, MR, AF and VN designed the study and supervised the experimental work. ES, CRS, FJPC, CC, AF and VN performed the experimental work. ES, CJC, and FJPC analyzed the data. FJPC, AF, ES and VN wrote the manuscript, with input from all authors. All authors read and approved the final manuscript

## Supplementary Material

Additional file 1 **List of genes differentially expressed by 2-fold in experimental Group A (CLA supplementation during gestation and during suckling through dam's milk)**. Excel file containing the list of 2-fold differentially expressed genes in group A with respect to the control (Group D) generated using GeneSpring software. It includes the gene symbol of all genes and the associated description. The absolute fold change values relative to the control group and the type of regulation (Up or Down) are provided. The differentially expressed transcripts corresponding to open reading frames, transcribed sequences, cDNA clones or hypothetical genes were removed. Click here for file

Additional file 2 **List of genes differentially expressed by 2-fold in experimental Group B (CLA supplementation during gestation and during suckling through oral gavage)**. Excel file containing the list of 2-fold differentially expressed genes in group B with respect to the control generated as described in Additional file 1. Click here for file

Additional file 3 **List of genes differentially expressed by 2-fold in experimental Group C (CLA supplementation during suckling through oral gavage)**. Excel file containing the list of 2-fold differentially expressed genes in group C with respect to the control generated as described in Additional file 1. Click here for file
